# Differential Effects of Exposure to Maternal Obesity or Maternal Weight Loss during the Periconceptional Period in the Sheep on Insulin Signalling Molecules in Skeletal Muscle of the Offspring at 4 Months of Age

**DOI:** 10.1371/journal.pone.0084594

**Published:** 2013-12-26

**Authors:** Lisa M. Nicholas, Janna L. Morrison, Leewen Rattanatray, Susan E. Ozanne, Dave O. Kleemann, Simon K. Walker, Severence M. MacLaughlin, Song Zhang, Malgorzata S. Martin-Gronert, Isabella C. McMillen

**Affiliations:** 1 Sansom Institute for Health Research, School of Pharmacy and Medical Sciences, University of South Australia, Adelaide, South Australia, Australia; 2 Discipline of Physiology, School of Molecular and Life Sciences, University of Adelaide, Adelaide, South Australia, Australia; 3 University of Cambridge Metabolic Research Laboratories, Wellcome Trust-MRC Institute of Metabolic Science, Addenbrooke's Hospital, Cambridge, United Kingdom; 4 Turretfield Research Centre, South Australian Research and Development Institute, Rosedale, South Australia, Australia; University of Southampton, United Kingdom

## Abstract

Exposure to maternal obesity before and/or throughout pregnancy may increase the risk of obesity and insulin resistance in the offspring in childhood and adult life, therefore, resulting in its transmission into subsequent generations. We have previously shown that exposure to maternal obesity around the time of conception *alone* resulted in increased adiposity in female lambs. Changes in the abundance of insulin signalling molecules in skeletal muscle and adipose tissue precede the development of insulin resistance and type 2 diabetes. It is not clear, however, whether exposure to maternal obesity results in insulin resistance in her offspring as a consequence of the impact of increased adiposity on skeletal muscle or as a consequence of the programming of specific changes in the abundance of insulin signalling molecules in this tissue. We have used an embryo transfer model in the sheep to investigate the effects of exposure to either maternal obesity or to weight loss in normal and obese mothers preceding and for one week after conception on the expression and abundance of insulin signalling molecules in muscle in the offspring. We found that exposure to maternal obesity resulted in lower muscle GLUT-4 and Ser 9 phospho-GSK3α and higher muscle GSK3α abundance in lambs when compared to lambs conceived in normally nourished ewes. Exposure to maternal weight loss in normal or obese mothers, however, resulted in lower muscle IRS1, PI3K, p110β, aPKCζ, Thr 642 phospho-AS160 and GLUT-4 abundance in the offspring. In conclusion, maternal obesity or weight loss around conception have each programmed specific changes on subsets of molecules in the insulin signalling, glucose transport and glycogen synthesis pathways in offspring. There is a need for a stronger evidence base to ensure that weight loss regimes in obese women seeking to become pregnant minimize the metabolic costs for the next generation.

## Introduction

More women in the developed world are entering pregnancy with a high body mass index (BMI) in the overweight or obese range [1-5]. Obese women are more insulin resistant than their normal weight counterparts, both before and during pregnancy [6] and this is associated with an increased risk of developing gestational diabetes mellitus (GDM) and of giving birth to a large baby with increased fat mass [6-9]. Exposure to either maternal obesity or to impaired glucose tolerance during pregnancy is associated with an increased risk of obesity and insulin resistance in childhood and adult life [10-12]. Additionally, experimental studies have investigated the impact of maternal high fat feeding on the postnatal metabolic phenotype of offspring. It has been shown that maternal high fat feeding leads to an increase in adiposity with [13] or without [14] absolute increases in body mass and poor glucose tolerance [14-17] and insulin resistance [15-17] in the offspring. 

A study by Long and colleagues found that exposure of ewes to a period of maternal overnutrition from before conception and throughout pregnancy resulted in an increase in offspring adiposity as well as a decrease in glucose tolerance and insulin sensitivity in adult offspring [18]. Moreover, these offspring also had decreased abundance of a specific subset of insulin signalling molecules in skeletal muscle [19]. These findings and other experimental studies suggest that exposure to maternal obesity may result in an ‘intergenerational cycle’ of obesity and insulin resistance [2,20,21].

As most women who are obese at conception remain obese through their pregnancy, it is difficult to determine the separate contributions of maternal pre-pregnancy BMI and glycaemic control on the metabolic outcomes for the offspring in human studies. We have previously used an embryo transfer model in the sheep to show that exposure of the oocyte and early embryo to maternal obesity during the period around the time of conception alone results in an increase in body fat mass in the female offspring which is ablated by a period of maternal dietary restriction and weight loss [22]. While maternal dieting before pregnancy may have metabolic benefits, there can be metabolic costs for the offspring. The Dutch famine study has shown that exposure to undernutrition during both early and mid-pregnancy in a population that was previously well nourished was associated with a reduction in glucose tolerance and increased insulin concentrations at age 50 and 58 [23]. Furthermore, experimental evidence in sheep has shown that maternal undernutrition during the periconceptional period has an adverse impact on the glucose-insulin axis of 10 month old post-natal lambs [24]. There is also evidence that this impaired glucose tolerance persists in the adult offspring [25]. Thus exposure to maternal obesity or to maternal dietary restriction in the periconceptional period are each associated with poor metabolic outcomes in the offspring, but the mechanisms by which these effects are transduced from mother to offspring are not understood. This is important as weight loss regimes in obese women to improve fertility, pregnancy outcomes and the metabolic health of her offspring are more feasible in the periconceptional period than during the rest of pregnancy.

Both clinical and experimental studies have shown that changes in the abundance of insulin signalling molecules in skeletal muscle and adipose tissue precede the development of insulin resistance and type 2 diabetes mellitus (T2DM) [26-29]. It is not known whether increased adiposity in offspring exposed to maternal obesity leads to insulin resistance as a result of the prevailing obesity [30,31] or whether exposure to maternal obesity programs specific changes in the abundance of insulin signalling molecules in insulin sensitive tissues [28,29,32] such as skeletal muscle. Furthermore it is not known whether exposure to maternal dietary restriction results in changes in different subsets of insulin signalling molecules within the skeletal muscle of the offspring. 

The rate-limiting step for glucose clearance in muscle is the transport of glucose across the plasma membrane by facilitated diffusion of glucose through a family of specific glucose transporters (GLUTs). GLUT-4 is actively translocated to the plasma membrane in response to insulin [33,34]. In the presence of insulin, insulin receptor (IR) phosphorylates insulin receptor substrate (IRS) proteins, which act as docking proteins for the activation of phosphatidylinositol 3-kinase (PI3K). PI3K catalyses the formation of phosphatidylinositol (3–5)-triphosphate (PIP_3_) which allows the activation of 3-phosphoinositide-dependent protein kinase 1 (PDK1) and Akt and atypical protein kinase C (aPKC) through phosphorylation of the Thr 308 and Thr 410 sites respectively [34]. The positive actions of PI3K can be negatively regulated by phospholipid phosphatases e.g. phosphatase and tensin homologue (PTEN) [35]. 

Activation of Akt phosphorylates and inhibits the Akt substrate of 160 kDa (AS160), which is involved in the regulation of glucose uptake through the redistribution of GLUT-4 from intracellular vesicles to the plasma membrane [36,37]. Similarly, aPKCs play a role in insulin-stimulated glucose uptake and GLUT4 translocation in adipocytes and muscle [33]. Furthermore, Akt is also involved in the regulation of glycogen synthesis through the actions of the serine/threonine kinase glycogen synthase kinase 3 (GSK3) which consists of two isoforms, GSK3α and GSK3β and which phosphorylates and inactivates glycogen synthase (GS) [35,38,39]. In resting cells, GSK3 activity is high but on stimulation, GSK3 is inactivated through phosphorylation; GSK3α is phosphorylated at Ser 21 and GSK3β at the equivalent residue, Ser 9 [38,39]. 

In the present study, we have used an embryo transfer model in the sheep, which was established by Rattanatray et al. [22] to investigate the effects of maternal obesity and of dietary restriction during the periconceptional period alone on the markers of insulin action in skeletal muscle of the pre-pubertal offspring at 4 months of age. Investigation of the changes that occur in the offspring would highlight the impact of the early nutritional environment on the programming of the insulin signalling pathway ahead of the development of frank insulin resistance and T2DM in later life. We have previously shown that exposure to maternal obesity and/or dietary restriction during the periconceptional period had no effect on plasma concentrations of glucose, insulin and non-esterified fatty acids (NEFA) in the offspring at four months of age [22]. We hypothesised that exposure to maternal obesity or to maternal dietary restriction and weight loss during the periconceptional period would have a differential impact on the expression of key molecules within the insulin signalling pathway in skeletal muscle of the offspring in postnatal life. 

## Materials and Methods

### Animals and nutritional feeding regime

All procedures were approved by the University of Adelaide Animal Ethics Committee and the Institute for Medical and Veterinary Science Animal Ethics Committee. Briefly, Merino ewes were weighed and body condition scores (BCS) assessed employing a 1.0 - 5.0 scale with 0.5 intervals [40,41]. During a 2 week acclimatisation period, ewes were fed a diet containing cereal hay, lucerne hay, barley, oats, almond shells, lupins, oat bran, lime and molasses (Johnsons & Sons Pty. Ltd., Kapunda, South Australia, Australia). The pellets provided 9.5 MJ/kg metabolizable energy and 120 g/kg crude protein and contained 90.6% dry matter. All ewes received 100% of nutritional requirements as defined by the Agricultural and Food Research Council[42]. 

### Donor ewes

Donor ewes (*n*=23) of normal body condition were then randomly assigned to one of 4 nutritional treatment groups, either control-control (CC), control-restricted (CR), high-high (HH) or high-restricted (HR) (Figure S1 in File S1).

1 CC ewes (n=6) were maintained at 100% metabolizable energy requirements (MER) [42] for 4 months before and 1 week after conception;
2 CR ewes (n=6) were maintained at 100% MER for the first 3 months, and then were placed on an energy restricted diet of 70% MER for 1 month before and 1 week after conception;
3 HH ewes (n=6) were fed an *ad* *libitum* diet (170-190% MER) for 4 months before and 1 week after conception; and
4 HR ewes (n=5) were fed an *ad* *libitum* diet (170-190% MER) for 3 months, and then were placed on an energy restricted diet of 70% MER for 1 month before and 1 week after conception;

Donor ewes were weighed and their BCS assessed approximately every 2 weeks after commencing the feeding regimen until embryo transfer at 6 - 7 days after conception. At conception and at embryo transfer, donor ewes in the HH and HR groups were significantly heavier than ewes in the CC and CR groups [22,43].

### Superovulation

The reproductive cycle of all experimental ewes was synchronized and super ovulation was induced as described previously [43]. 

### Artificial insemination and embryo collection

Fresh semen was collected as previously described [44]. Donor ewes were inseminated with ~ 2 x 10^7^ spermatozoa placed into each uterine horn 36 h after pessary withdrawal. Embryos were collected by laparotomy (Baxter, Old Toongabbie, NSW, Australia) 6 - 7 days after artificial insemination. General anaesthesia was induced in the ewe by an intravenous injection of sodium thiopentone (1.25 g, Pentothal, Rhone Merieux, Pinkenba, Qld, Australia) and maintained with 2.5-4% halothane inhalation anaesthetic (Fluothane, ICI, Melbourne, Vic, Australia) in oxygen. Embryos were held at 38.5°C in HEPES-buffered synthetic oviduct fluid supplemented with bovine serum albumin and amino acids. Maternal obesity and/or dietary restriction during the periconceptional period had no impact on the number of embryos collected or on the developmental stage of the collected embryos (Table 1). 

**Table 1:** The number of embryos recovered during embryo collection at 1 week after conception and the proportion of embryos observed in the compact morula, early blastocyst, blastocyst and expanded blastocyst stage from in normal weight ewes (CC), normal weight ewes put on a dietary restriction regime for one month before and one week after conception (CR), overnourished, obese ewes (HH) and obese ewes put on a dietary restriction regime for one month before and one week after conception (HR).

	**Compact morula**	**Early blastocyst**	**Blastocyst**	**Expanded blastocyst**
**CC (*n*=55)**	54.5%	9.1%	20.0%	16.4%
**CR (*n*=45)**	57.8%	15.5%	20.0%	6.7%
**HH (*n*=42)**	42.9%	7.1%	47.6%	2.4%
**HR (*n*=55)**	45.4%	10.9%	27.3%	16.4%

### Recipient ewes

A total of 198 embryos were recovered from the 23 donor ewes. 62 recipient ewes were selected to ensure the birth of at least 8 offspring per treatment. Thus only 62 donor embryos were required for transfer. Donor embryos of good quality were recovered and transferred to synchronized recipient ewes maintained on a control diet (100% MER). The embryos that were not transferred were either frozen or disposed of. There was no difference in the weights of the recipient ewes allocated to carry the CC, CR, HH or HR embryos [22,43]. Each recipient ewe received only one embryo, resulting in 4 treatment groups, i.e. CC, n = 13; CR, n = 16; HH, n = 17; and HR, n = 16. These ewes were fed a control diet for the remainder of pregnancy through to weaning [42]. Pregnancy was confirmed at 49 days gestation in 47 singleton pregnancies (Table 2). Lambs were delivered naturally (term = 150±3 d). One lamb was excluded from the study because it had an accident to its leg after birth and failed to feed and gain weight. Another lamb was born with a congenital malformation and was euthanized shortly after birth.

**Table 2:** Pregnancy outcomes and the survival of lambs of normal weight ewes (CC), normal weight ewes put on a dietary restriction regime for one month before and one week after conception (CR), overnourished, obese ewes (HH) and obese ewes put on a dietary restriction regime for one month before and one week after conception (HR).

	**Embryos transferred**	**Number of positive pregnancy scans**	**Pregnancy rate (%)**	**Live births**	**Number of lambs which survived to 4 months of age**	
					**Males**	**Females**
**CC**	13	9	69	8	2	5
**CR**	16	12	75	10	7	3
**HH**	17	13	76	12	7	5
**HR**	16	13	75	13	5	7

Lambs [CC, n = 7 (Males: n=2, Females: n=5); CR, n = 10 (Males: n=7, Females: n=3); HH, n = 12 (Males: n=7, Females: n=5); and HR, n = 12 (Males: n=5, Females: n=7)] were weaned at 3 months of age after which they were housed in individual pens and fed a control diet (100% MER). Nutritional treatment had no effect on live birth rate (CC, 62%; CR, 63%; HH, 71%; and HR, 81%). Furthermore, there was no effect of nutritional treatment on either the birth weight or body weight of lambs at 4 months. Male lambs were significantly heavier at birth and at 4 months compared to females independent of the nutritional treatment during the periconceptional period [22,43]. 

### Post mortem and tissue collection

At 4 months, lambs were fasted and killed with an overdose (~ 30 mg/kg iv) of sodium pentobarbitone (Virbac Pty. Ltd., Peakhurst, NSW, Australia). A sample of quadricep muscle was snap frozen in liquid nitrogen and stored at -80°C. 

### Quantification of mRNA expression using quantitative real-time RT-PCR

RNA was extracted from skeletal muscle (CC, n = 7; CR, n = 10; HH, n = 12; and HR, n = 12) using TRIzol reagent (Invitrogen, Groningen, Netherlands) and purified using RNeasy Mini Kit (Qiagen, Basel, Switzerland). cDNA was synthesized using 1ug RNA by reverse transcription using Superscript III (Invitrogen Australia Pty Ltd, Mount Waverley, Victoria, Australia). Negative controls containing no RNA or Superscript III were used to test for DNA contamination.

The relative expression of mRNA transcripts of IR, GLUT-4 and acidic ribosomal protein large subunit P0 (RPLP0) (Table S1 in File S1) in skeletal muscle were measured by quantitative real time reverse transcription-PCR (qRT-PCR) using the Sybr Green system in an ABI Prism 7500 Sequence Detection System (PE Applied Biosystems, Foster City, CA, USA). 

Each amplicon was sequenced to ensure authenticity of the DNA product and qRT-PCR melt curve analysis performed to demonstrate amplicon homogeneity. Each qRT-PCR reaction well contained: 6 µl Sybr Green Master Mix (PE Applied Biosystems, Foster City, CA), 1 µl primer, 2 µl molecular grade H_2_O and 1 µl of cDNA (50 ng µl^-1^). Controls containing no reverse transcriptase were also used. The cycling conditions consisted of 40 cycles at 95°C for 15 sec and at 60°C for 1 min.

The abundance of each mRNA transcript was measured and expression relative to RPLP0 calculated using the comparative threshold cycle (C_t_) method (*Q-Gene* analysis software), which provides a quantitative measure of the relative abundance of a specific transcript in different tissues, which takes into account differences in the amplification efficiencies of the target and reference (RPLP0) genes. The C_t_ value was taken as the lowest statistically significant (>10 _SD_) increase in fluorescence above the background signal in an amplification reaction. 

### Quantification of insulin signaling protein abundance by Western blotting

The protein abundance of the insulin signaling molecules was determined using Western blotting as described in detail elsewhere [45]. Briefly, tissue samples (150 mg) (CC, n = 6, 2 males & 3 females; CR, n = 6, 3 males & 3 females; HH, n = 6, 3 males & 3 females; and HR, n = 6, 3 males & 3 females) were homogenized in lysis buffer [50 mmol/l HEPES (pH 8), 150 mmol/l sodium chloride, 1% Triton X100, 1 mmol/l sodium orthovanadate, 30 mmol/l sodium fluoride, 10 mmol/l sodium pyrophosphate, 10 mmol/l EDTA and a protease inhibitor cocktail] and centrifuged at 15 000 *g* at 4°C for 5 min to remove lipid and insoluble material. Protein content of the clarified extracts was determined by modification of the Lowry method. Equal volumes of protein (10 µg) were subjected to SDS-PAGE. The proteins were transferred to polyvinylidene diflouride membrane (Millipore, MA, USA), blocked overnight and then incubated with primary antisera raised against: IRβ subunit (IR_B_) and GLUT-4 (Abcam, Cambridge, UK), IRS1 and PI3K p85α subunit (Upstate Biotechnology, Millipore, Billerica, USA), Tyr 1162/1163 phospho-IR, PI3K p110β subunit and aPKCζ (Santa Cruz Biotechnology, Santa Cruz, USA) and PDK1, Ser 241 phospho-PDK1, PTEN, Akt1, Akt2, Ser 473 phospho-Akt, Thr 308 phospho-Akt, AS160, Thr 642 phospho-AS160, GSK3α, Ser 21 phospho-GSK3α, GSK3β, Ser 9 phospho-GSK3β, glycogen synthase (GS) and Ser 641 phospho-GS (Cell Signalling Technology, Danvers, USA) [45]. Membranes were washed and bound antibody detected using horseradish peroxidase-conjugated secondary antibodies and enhanced chemiluminescence reagents according to the manufacturer’s instructions (Thermo Scientific, Rockford, IL, USA). AlphaEaseFC^TM^ software (Alpha Innotech Corporation, CA, USA) was used to quantify the density of specific bands. To monitor the linearity of the density measurements, 10 µg and 20 µg of the same protein sample was loaded onto each gel to confirm that the chemiluminescent signal changed in a linear manner. Prior to Western blotting analysis, samples (20 µg protein) were subjected to SDS-PAGE and gel was stained with Coomassie Brilliant Blue (Thermo Scientific, Waltham, MA, USA) and there were no differences in abundance of the major proteins in samples between the different experimental groups. 

### Statistical analysis

Data are presented as mean ± SEM. The effects of periconceptional nutrition and gender on the levels of mRNA expression and protein abundance in skeletal muscle of lambs at 4 months of age were determined using a two-way ANOVA (SPSS for Windows version 18; SPSS Inc., Chicago, IL) with donor ewe number nested within nutritional treatment groups to identify lambs arising from the same donor. When there was an interaction between the effects of periconceptional nutrition and gender, the effect of periconceptional nutrition was determined separately in males and females. The Duncan’s post hoc test was used to determine significant differences between groups and a probability level of 5% (P<0.05) was taken as significant. 

## Results

### Effects of maternal obesity during the periconceptional period (HH) on expression of insulin signalling molecules and molecules involved in glucose transport in skeletal muscle of postnatal lambs

**Figure 1:**
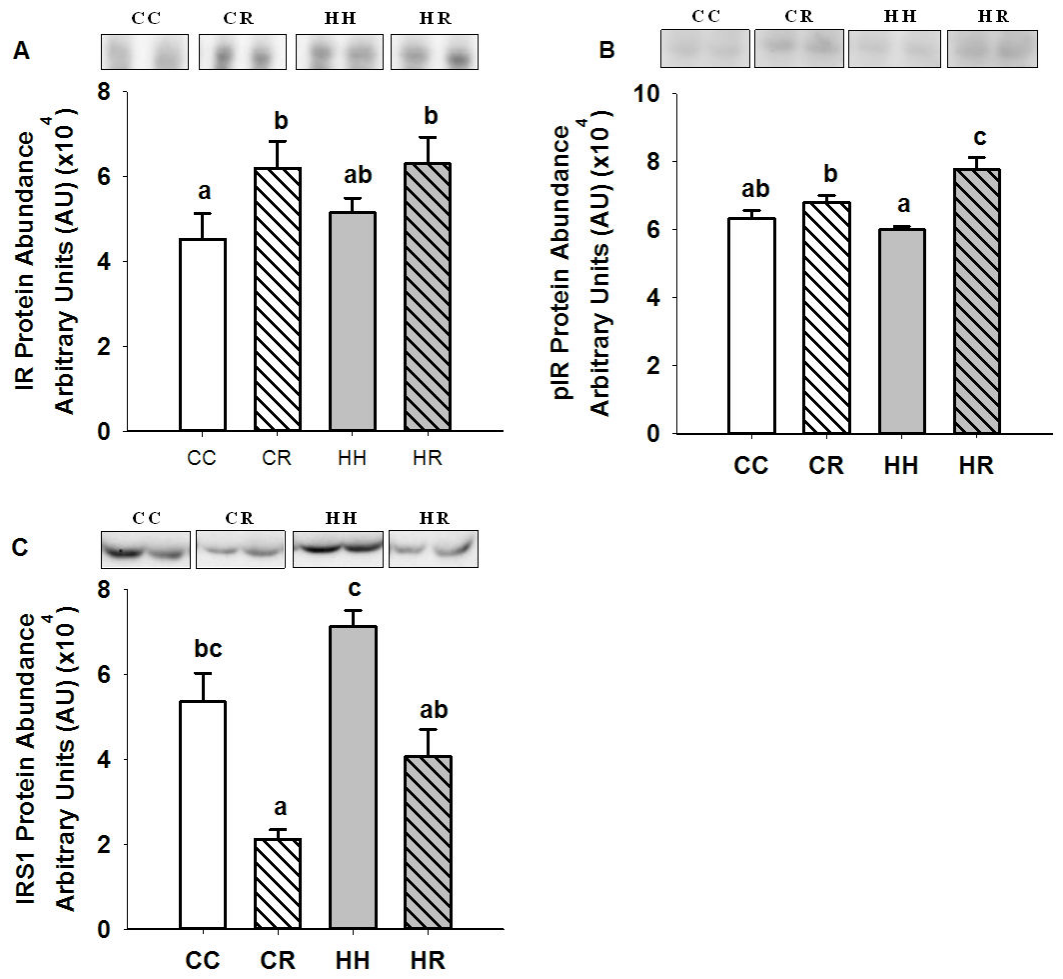
Skeletal muscle IR, Tyr 1162/1163 phosphoIR and IRS1 protein abundance in four month old lambs of normal weight ewes (CC), normal weight ewes put on a dietary restriction regime for one month before and one week after conception (CR), overnourished, obese ewes (HH) and obese ewes put on a dietary restriction regime for one month before and one week after conception (HR). Different superscripts denote mean values that are significantly different. *n*=6 lambs, CC, 2 males, 3 females; CR, HH & HR, 3 males, 3 females. All data are mean ± s.e.m.

**Figure 2:**
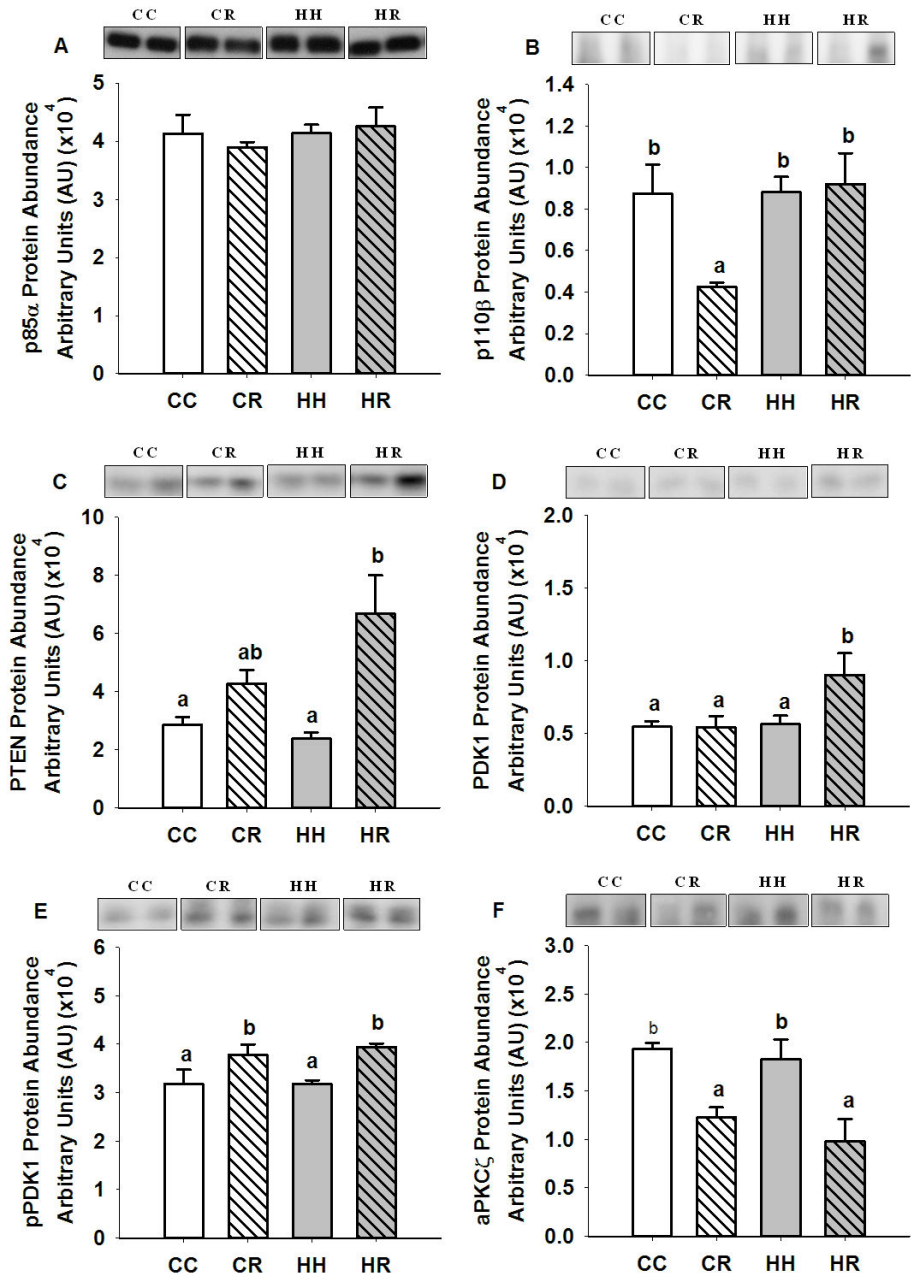
Skeletal muscle p85α, p110β, PTEN, PDK1, Ser 241 phosphoPDK1 and aPKCζ protein abundance in four month old lambs of normal weight ewes (CC), normal weight ewes put on a dietary restriction regime for one month before and one week after conception (CR), overnourished, obese ewes (HH) and obese ewes put on a dietary restriction regime for one month before and one week after conception (HR). Different superscripts denote mean values that are significantly different. *n*=6 lambs, CC, 2 males, 3 females; CR, HH & HR, 3 males, 3 females. All data are mean ± s.e.m.

**Figure 3:**
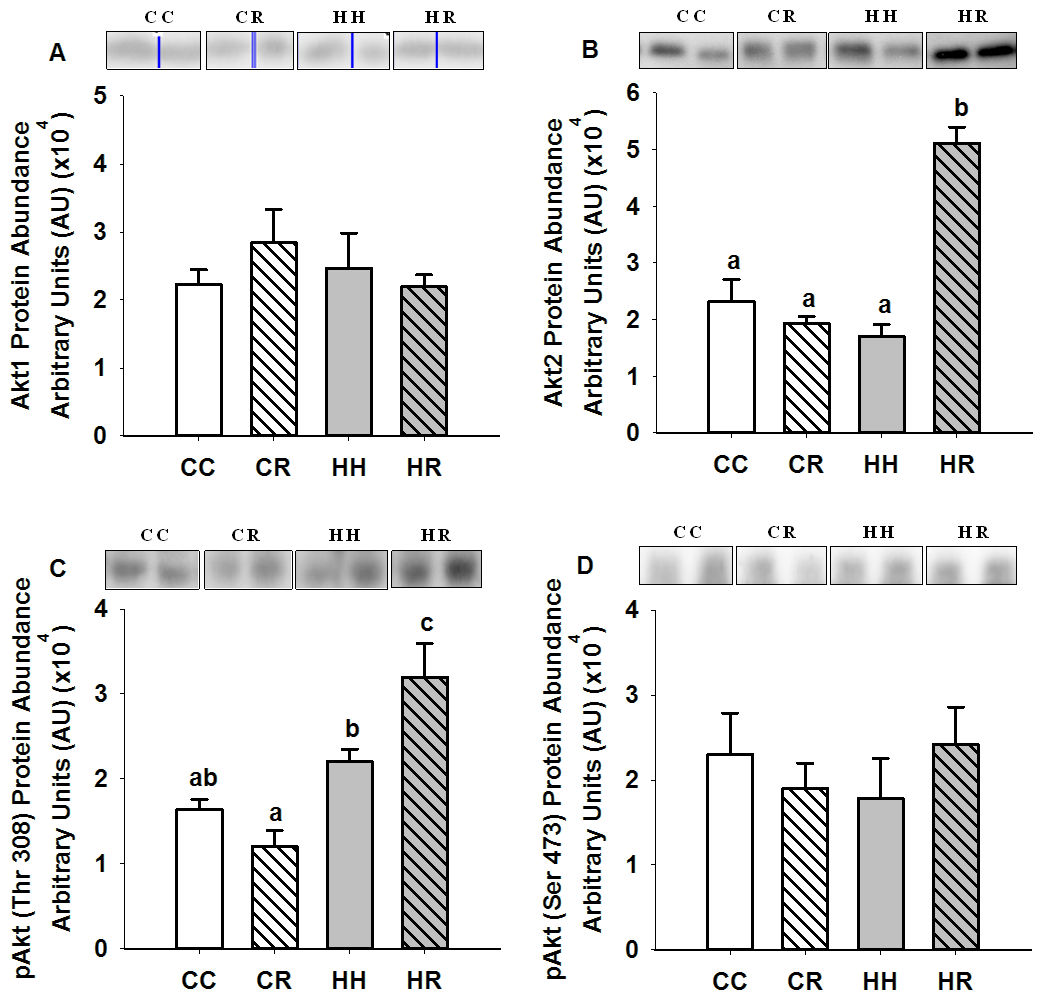
Skeletal muscle Akt1, Akt2, Thr 308 and Thr 473 phosphoAkt protein abundance in four month old lambs of normal weight ewes (CC), normal weight ewes put on a dietary restriction regime for one month before and one week after conception (CR), overnourished, obese ewes (HH) and obese ewes put on a dietary restriction regime for one month before and one week after conception (HR). Different superscripts denote mean values that are significantly different. *n*=6 lambs, CC, 2 males, 3 females; CR, HH & HR, 3 males, 3 females. All data are mean ± s.e.m.

There was no difference in the mRNA expression (CC, 0.16 ± 0.01; CR, 0.13 ± 0.02; HH, 0.19 ± 0.03; HR, 0.17 ± 0.01) and protein abundance of IR and protein abundance of Tyr 1162/1163 phospho-IR, IRS1 (Figures 1a - c), the p85α and p110β subunits of PI3K, PTEN, PDK1, Ser 241 phospho-PDK1, aPKCζ (Figures 2a - f), Akt1, Akt2, Thr 308 and Ser 473 phospho-Akt (Figures 3a - d) in skeletal muscle between the HH and CC lambs. 

**Figure 4:**
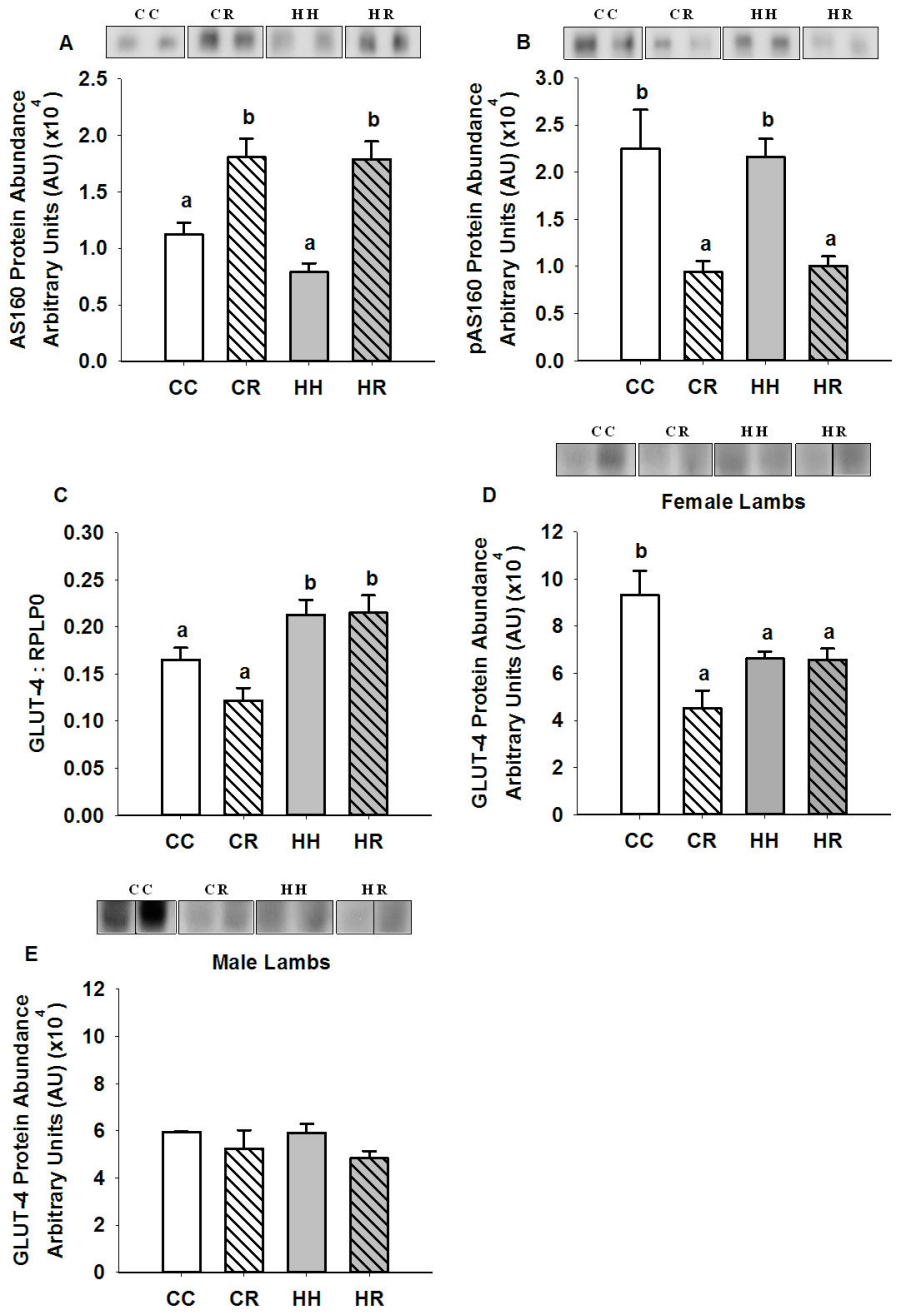
Skeletal muscle AS160, Thr 642 phospho-AS160 and GLUT-4 abundance in four month old lambs of normal weight ewes (CC), normal weight ewes put on a dietary restriction regime for one month before and one week after conception (CR), overnourished, obese ewes (HH) and obese ewes put on a dietary restriction regime for one month before and one week after conception (HR). Different superscripts denote mean values that are significantly different. *n*=6 lambs, CC, 2 males, 3 females; CR, HH & HR, 3 males, 3 females (Western blotting) CC, *n* = 7; CR, *n* = 10; HH, *n* = 12; and HR, *n* = 12 lambs (qRT-PCR). All data are mean ± s.e.m.

While the abundance of AS160 and Thr 642 phospho-AS160 protein in the skeletal muscle of the HH lambs was not different to that in the CC lambs (Figures 4a & b), GLUT-4 mRNA expression was higher (P<0.05) in the HH compared to the CC group (Figured 4c) while GLUT-4 abundance was decreased (P<0.05) in the HH female (Figure 4d) but not male (Figure 4e) lambs, when compared to the CC group. 

### Effects of maternal obesity during the periconceptional period on molecules involved in glycogen synthesis in skeletal muscle of postnatal lambs

**Figure 5:**
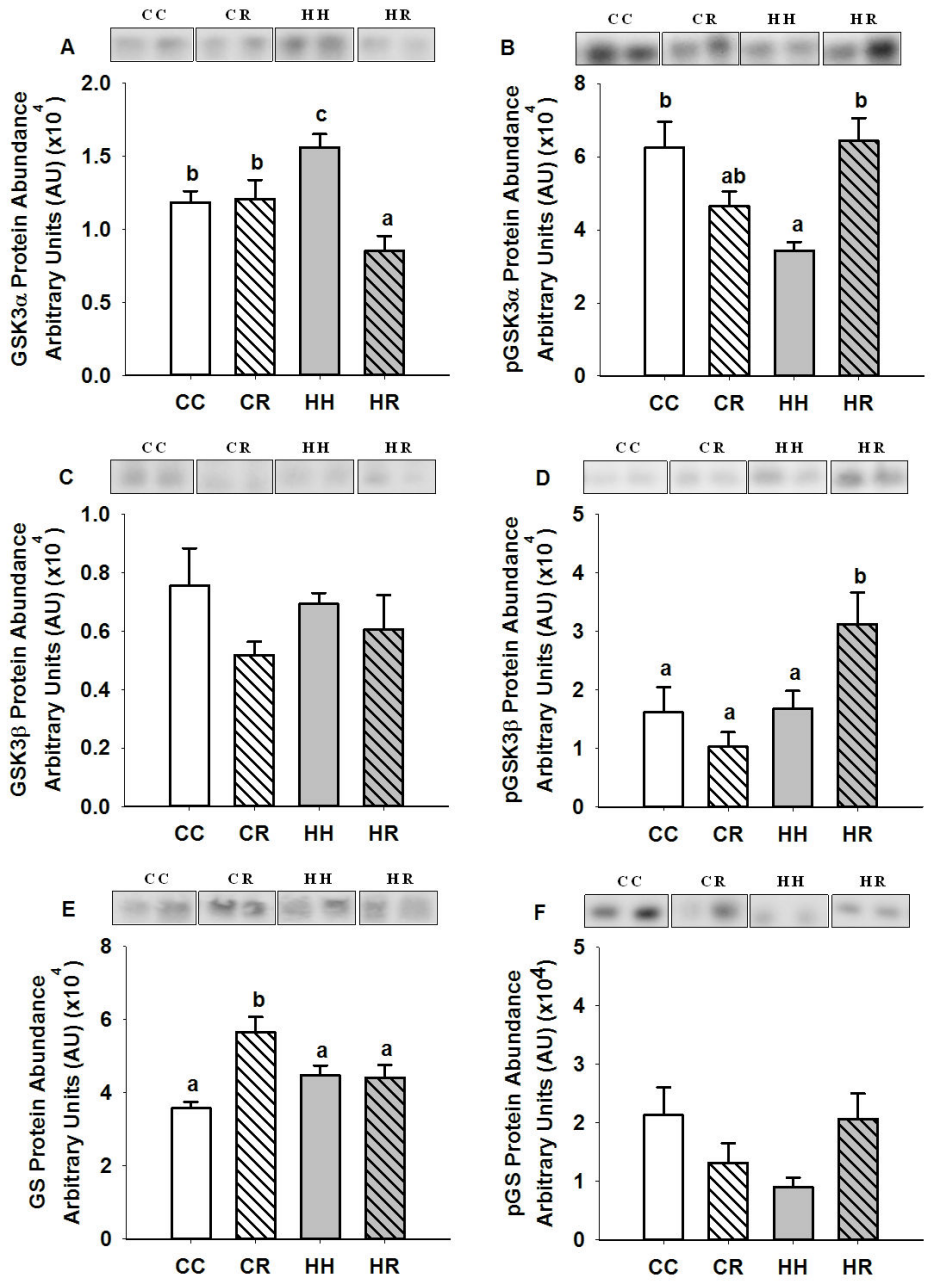
Skeletal muscle GSK3α, Ser 21 phospho-GSK3α, GSK3β, Ser 9 phospho-GSK3β, GS and Ser 641 phospho-GS protein abundance in four month old lambs of normal weight ewes (CC), normal weight ewes put on a dietary restriction regime for one month before and one week after conception (CR), overnourished, obese ewes (HH) and obese ewes put on a dietary restriction regime for one month before and one week after conception (HR). Different superscripts denote mean values that are significantly different. *n*=6 lambs, CC, 2 males, 3 females; CR, HH & HR, 3 males, 3 females. All data are mean ± s.e.m.

The abundance of GSK3α was higher (P<0.05) while the abundance of Ser 21 phospho-GSK3α was lower (P<0.05) in the skeletal muscle of the HH compared to the CC lambs (Figure 5a-b). There was no difference, however, in the abundance of GSK3β, Ser 9 phospho- GSK3β, GS or Ser 641 phospho-GS in the skeletal muscle between the HH and CC lambs (Figures 5c - f).

Effects of dietary restriction in normal weight and obese ewes during the periconceptional period on expression of insulin signalling molecules and molecules involved in glucose transport in skeletal muscle of postnatal lambs

The expression of IR mRNA in skeletal muscle was not different in either the CR or HR groups when compared to the CC lambs (CC, 0.16 ± 0.01; CR, 0.13 ± 0.02; HH, 0.19 ± 0.03; HR, 0.17 ± 0.01). The abundance of IR protein in muscle was higher (P<0.05), however in the CR and HR groups when compared to the CC group (Figure 1a). The abundance of Tyr 1162/1163 phospho-IR abundance was also higher (P<0.01) in the HR, but not the CR group compared to the CC group (Figure 1b). The abundance of IRS1 in muscle was lower (P<0.01) in the CR when compared to the CC lambs (Figure 1c). 

There was no difference in the abundance of p85α in skeletal muscle of lambs in the CR and HR groups (Figure 2a). In contrast, p110β expression was lower (P<0.05) in the CR compared to the CC group (Figure 2b). The abundance of PTEN, however, was increased (P<0.01) in the HR group (Figure 2c). Downstream of PI3K, PDK1 abundance was increased (P<0.05) in the HR group and Ser 241 phospho-PDK1 abundance was increased (P<0.01) in both the CR and HR groups compared to CC lambs (Figures 2d & e). In contrast, aPKCζ protein abundance was decreased (P<0.05) in the skeletal muscle of these CR and HR lambs (Figure 2f). 

Maternal dietary restriction in the periconceptional period had no effect on Akt1 and Ser 473 phospho-Akt protein abundance but the abundance of Akt2 and Thr 308 phospho-Akt was increased (P<0.01) in skeletal muscle of the HR offspring (Figures 3a - d). 

AS160 protein abundance was higher (P<0.01) and Thr 642 phospho-AS160 abundance (P<0.01) was lower in skeletal muscle of the CR and HR offspring when compared to controls (Figure 4a & b). GLUT-4 mRNA expression was increased (P<0.05) in skeletal muscle of lambs in the HR but not the CR group (Figure 4c). GLUT-4 protein abundance was decreased (P<0.05) however, in female (Figure 4d) but not male (Figure 4e) lambs in both the CR and HR groups compared to the CC group. 

Effects of dietary restriction during the periconceptional period in normal weight and obese ewes on expression of molecules involved in glycogen synthesis in skeletal muscle of postnatal lambs

GSK3α protein abundance was decreased (P<0.05) in skeletal muscle of lambs in the HR but not the CR group when compared to the CC group (Figure 5a). There was, however, no difference in Ser 21 phospho-GSK3α abundance between lambs in the HR, CR and CC groups (Figure 5b). GSK3β protein abundance was also not different between these lambs (Figure 5c). In contrast, Ser 9 phospho-GSK3β abundance was increased (P<0.05) in skeletal muscle of HR but not CR lambs (Figure 5d). There was also an increase (P<0.01) in GS protein abundance but only in the CR group compared to the CC group whereas Ser 641 phospho-GS abundance was not different between the CR, HR and CC lambs (Figures 5e & f).

## Discussion

Epidemiological [10,11] and experimental [13-17] studies have shown that exposure to maternal obesity before and throughout pregnancy increase the risk of obesity and insulin resistance in the offspring [2,20,21]. It is not clear, however, whether exposure to maternal obesity results in insulin resistance in her offspring as a consequence of the impact of increased adiposity in insulin sensitive tissues [30,31] or as a consequence of the programming of changes in the abundance of insulin signalling molecules in these tissues [28,29,32]. There have also been no studies which have investigated whether exposure to maternal obesity only during the period around conception results in the programming of the insulin signalling pathway in skeletal muscle. We have used an embryo transfer model in the sheep, which was established by Rattanatray et al. [22] to investigate the effects of exposure to either maternal obesity and/or dietary restriction before and for one week after conception on the markers of insulin action in skeletal muscle of the offspring. Rattanatray et al. have previously found that that there was a gender-specific effect of maternal obesity specifically during the periconceptional period on the body fat mass of lambs at four months of age [22]. Female but not male lambs were found to have increased total fat mass [22]. Specifically, the greatest impact of maternal periconceptional obesity appeared to be on the visceral fat depots i.e. the perirenal and omental fat depots in these female lambs [22]. 

### The impact of maternal obesity during the periconceptional period on expression of insulin signalling molecules and molecules involved in glucose transport in skeletal muscle of postnatal lambs

**Figure 6:**
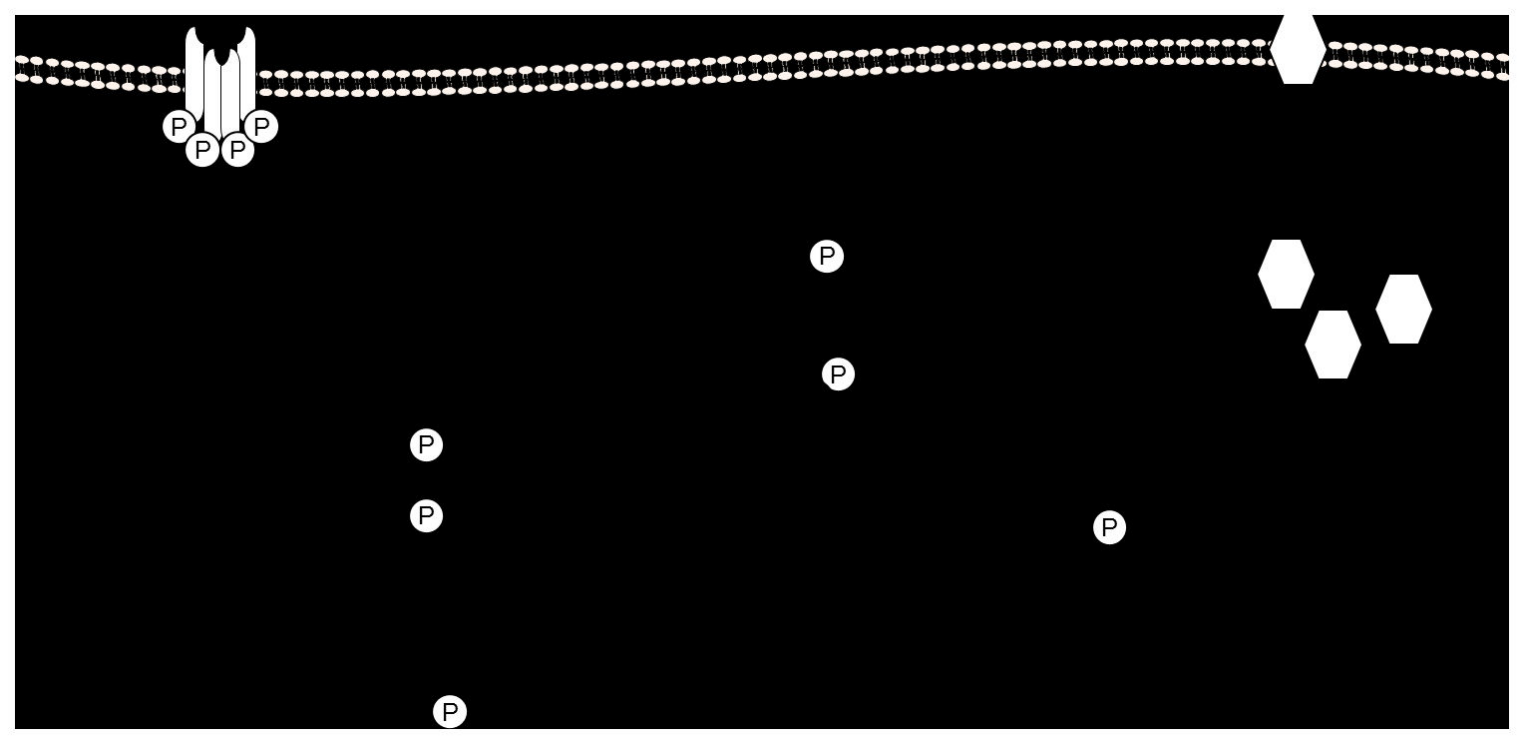
The impact of maternal obesity during the periconceptional period on expression of insulin signalling molecules and molecules involved in glucose transport and glycogen synthesis in skeletal muscle of postnatal lamb.

We have demonstrated that exposure to maternal obesity during the periconceptional period had no effect on the abundance of a number of insulin signalling molecules in skeletal muscle of lambs at four months including IR and IRS1. There was also no change in the abundance of both the regulatory and catalytic subunits of PI3K as well as its negative regulator, PTEN or in the abundance of total and phosphorylated PDK1 and Akt (Figure 6). 

Interestingly, however, exposure to maternal obesity during the periconceptional period resulted in higher GLUT-4 mRNA expression and lower GLUT-4 protein abundance in skeletal muscle (in female lambs). Defects in GLUT 4 expression as well as impairment of GLUT-4 recruitment to the plasma membrane have been implicated in the onset of insulin resistance and type 2 diabetes [46]. It is possible, that the increase in skeletal muscle GLUT-4 mRNA expression is a compensatory response to the decrease in abundance of GLUT-4, although this decrease was only significant in female HH lambs. Interestingly, Camps and colleagues found that rats with streptozotocin-induced diabetes had a similar mismatch between the expression and abundance of GLUT-4 in skeletal muscle [47]. The fact that changes in GLUT-4 protein abundance has been found in female but not male lambs raises the possibility that its abundance is associated with the increased fat mass observed in female lambs.

In the present study, there was no evidence for a change in expression or abundance of aPKCζ, AS160 and Thr 642 phospho-AS160 in the muscle of HH lambs. Kern and colleagues have previously shown that the amount of GLUT-4 protein is the primary factor in determining the maximal rate of glucose transport into skeletal muscle [48] suggesting that female HH lambs may be at risk of a decrease in muscle glucose uptake [49]. At four months of age, however, there were no differences in plasma insulin, glucose and NEFA concentrations in the lambs from different treatment groups [22]. 

### The impact of maternal obesity during the periconceptional period on expression of molecules involved in glycogen synthesis in skeletal muscle of postnatal lambs

Insulin activates GS by promoting its de-phosphorylation at a cluster of serine residues. Under basal conditions, these residues remain phosphorylated through the actions of GSK3 [38]. Upon activation of the insulin signalling cascade, however, GSK3 is inactivated by the actions of Akt, which phosphorylates Ser 9 on GSK3α and Ser 21 on GSK3β [39]. GSK3 therefore serves as a negative modulator of insulin action on GS [38]. In our study, maternal obesity during the periconceptional period resulted in an increase in GSK3α and a decrease in Ser 9 phospho-GSK3α abundance in the absence of a change in phosphorylated Akt. These changes would be expected to result in an increase in GSK3 and a consequent inhibition of the actions of insulin on GS. Elevated GSK3 levels are present in skeletal muscle in rodent models of obesity and T2DM [50,51] as well as in obese and diabetic humans [52]. Thus, exposure to maternal obesity during the periconceptional period appears to have relatively minimal impact on the early components of the insulin signalling pathway and a greater impact on the abundance of molecules downstream of Akt; GLUT 4 and GSK3α in skeletal muscle in the offspring (Figure 6). We also note that there was no correlation between either gene expression or protein abundance of the insulin signalling molecules and the total fat mass in either male or female lambs. 

The impact of maternal dietary restriction during the periconceptional period in normal weight and obese ewes on expression of insulin signalling molecules and molecules involved in glucose transport in skeletal muscle of postnatal lamb 

CR and HR lambs had higher abundance of IR protein and there was also a parallel increase in Tyr 1162/1163 phospho-IR in HR lambs. IRS1 abundance was, however, lower in the skeletal muscle of CR and to a lesser extent, HR lambs. The higher IR abundance may be a compensatory response in the context of decreased insulin signalling downstream of the receptor. There was also a lower abundance of the catalytic PI3K subunit, p110β in the CR lambs. Interestingly, the abundance of p110β has also been found to be lower in the skeletal muscle in individuals with a low birth weight (29). The decrease in skeletal muscle p110β in lambs in the face of unchanged p85α abundance may be important as p85α is a negative regulator of PI3K [53] when expressed at relatively higher levels [54]. In our study, the HR offspring also had increased abundance of the negative regulator of insulin signalling, PTEN. 

Downstream of PI3K, phosphorylation and thus activation of Ser 241 PDK1 is required for the subsequent activation of aPKCζ and Akt through phosphorylation of the Thr 410 and Thr 308 sites respectively [34]. There was an increase in PDK1 abundance in HR lambs and also an increased abundance of Ser 241 phospho-PDK1 in skeletal muscle of both CR and HR lambs indicating that irrespective of the total amount of PDK1 in the cell, exposure to dietary restriction during the periconceptional period results in enhanced activation of PDK1. 

Interestingly, while PDK1 activation appeared increased, there was a decrease in the abundance of aPKCζ in the muscle of CR and HR lambs. Together, these changes indicate that there is a compensatory increase in Ser 241 phospho-PDK1 abundance in the face of decreased aPKCζ in order to maintain aPKCζ activation. Decreased activation of aPKCζ has been shown to result in a decrease in insulin-stimulated glucose uptake and GLUT-4 translocation in skeletal muscle [33] and has also been observed in skeletal muscle of type-2 diabetic humans and rodents [55]. Furthermore, a decrease in the total abundance of aPKCζ has also been observed in muscle of early growth-restricted rat offspring [29]. There was also an increase in AS160 and a decrease in Thr 642 phospho-AS160 abundance in skeletal muscle of the CR and HR offspring. GLUT-4 mRNA expression was also decreased in CR lambs and there was lower GLUT-4 abundance in female lambs in the CR and HR groups.

**Figure 7:**
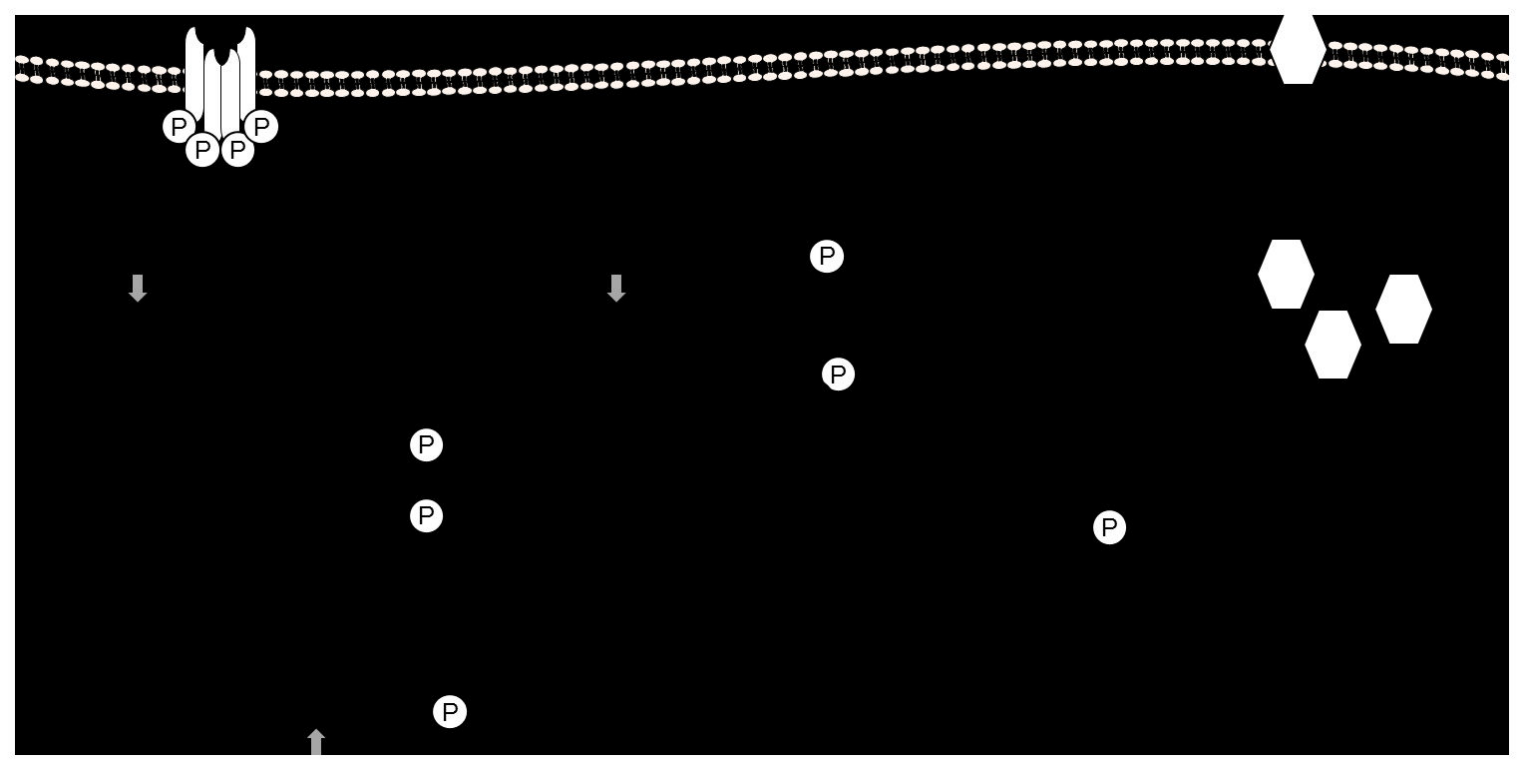
The impact of maternal dietary restriction during the periconceptional period in normal weight and obese ewes on expression of insulin signalling molecules and molecules involved in glucose transport and glycogen synthesis in skeletal muscle of postnatal lamb. Open arrows represent effects in CR lambs only, grey arrows represent effects in HR lambs only and filled arrows represent effects in both CR and HR lambs.

In summary, although there were no changes in plasma insulin, glucose and NEFA concentrations in these lambs at four months of age [22], our findings indicate that there may be a predisposition to insulin resistance in the skeletal muscle of offspring exposed to maternal dietary restriction in the periconceptional period. Interestingly, there appears to be a compensatory response to increase insulin signalling in skeletal muscle of lambs of obese ewes exposed to dietary restriction during the periconceptional period. Specifically, Akt2 and Thr 308 phospho-Akt abundance are higher in HR but not CR lambs (Figure 7).

The impact of maternal dietary restriction during the periconceptional period in normal weight and obese ewes on expression of molecules involved in glycogen synthesis in skeletal muscle of postnatal lambs

Whilst exposure to maternal obesity during the periconceptional period led to an increase in GSK3α protein abundance, in contrast, dietary restriction in obese ewes resulted in decreased GSK3α abundance in the offspring. Furthermore, HR lambs also had increased Ser 21 phospho-GSK3β abundance. Interestingly, offspring of normal weight ewes that were exposed to maternal dietary restriction during the periconceptional period had a higher abundance of GS in skeletal muscle.

Taken together, these results suggest that factors, which regulate components of the glycogen synthesis pathway, are sensitive to maternal body weight at the time of conception rather than to maternal weight loss during the periconceptional period. Although the abundance of different molecules were affected in skeletal muscle of CR (GS) compared to HR (GSK3α and Ser 21 phospho-GSK3β) lambs, maternal dietary restriction in both normal weight and obese ewes appeared to result in an enhanced capacity for glycogen synthesis in the skeletal muscle of the CR and HR offspring.

## Conclusions

We have shown that exposure to maternal obesity during the periconceptional period does not appear to impact directly on the early components of the insulin receptor signalling pathway in skeletal muscle of the offspring but rather results in specific changes to molecules downstream of Akt, namely GLUT-4 and GSK3α (Figure 6). These findings suggest that changes which emerge in the proximal components of the insulin signalling pathway in obese offspring of obese mothers may be a consequence of the prevailing obesity. It has been shown that elevated levels of GSK3 are present in skeletal muscle in rodent models of obesity and T2DM [50,51] as well as in obese and type 2 diabetic humans [52]. Thus exposure to maternal obesity from before and for only one week after conception may result in the programming of changes in skeletal muscle which may explain, in part, the vulnerability of offspring of obese mothers to T2DM.

Importantly, we have also shown that dietary restriction in either obese or normal weight mothers resulted in marked changes in the abundance of molecules in the early part of the insulin signalling pathway in skeletal muscle (Figure 7) and also that not all of the negative effects of maternal obesity during the periconceptional period on glucose uptake and glycogen synthesis were ablated by dietary restriction. These findings are of particular importance in the context of current clinical practice whereby dietary restriction is recommended to overweight and obese women who are seeking to become pregnant. 

Thus, the present study highlights that exposure of the oocyte and embryo to signals of maternal obesity and weight loss result in long term and persistent changes in the abundance of specific molecules involved in insulin signalling, glucose transport and glycogen synthesis in skeletal muscle of the offspring. This suggests that identification of the epigenetic mechanisms that are recruited within the developing embryo in the face of maternal obesity or weight loss, which result in the programming of the metabolic pathways identified in the present study, would be important to inform dietary intervention regimes in overweight or obese women seeking to become pregnant. 

## Supporting Information

File S1 Figure S1, Donor ewes were randomly allocated to 1 of 4 treatment groups and fed according to the nutritional treatment protocol from 5 months prior to conception. At 6-7d after conception, single embryos were transferred by laparoscopy into adult recipient ewes of normal weight. Ewes lambed normally and post mortem was conducted on the offspring at 4 months of age during which skeletal muscle samples were collected. Table S1, Oligonucleotide primer sequences for qRT-PCR analysis of IR, GLUT-4 and reference genes in skeletal muscle of postnatal lambs.
(DOCX)
