# Supplementary material for: Differential Effects of Exposure to Maternal Obesity or Maternal Weight Loss during the Periconceptional Period in the Sheep on Insulin Signalling Molecules in Skeletal Muscle of the Offspring at 4 Months of Age
Source: PLoS One. 2013 Dec 26;8(12):e84594. doi: 10.1371/journal.pone.0084594 (PMC3873457; doi:10.1371/journal.pone.0084594)
Supplement: File S1 — Figure S1, Donor ewes were randomly allocated to 1 of 4 treatment groups and fed according to the nutritional treatment protocol from 5 months prior to conception. At 6-7d after conception, single embryos were transferred by laparoscopy into adult recipient ewes of normal weight. Ewes lambed normally and post mortem was conducted on the offspring at 4 months of age during which skeletal muscle samples were collected. Table S1, Oligonucleotide primer sequences for qRT-PCR analysis of IR, GLUT-4 and reference genes in skeletal muscle of postnatal lambs. (DOCX) [file pone.0084594.s001.docx]

**Figure S1in File S1: Donor ewes were randomly allocated to 1 of 4 treatment groups and fed according to the nutritional treatment protocol from 5 months prior to conception. At 6-7d after conception, single embryos were transferred by laparoscopy into adult recipient ewes of normal weight (22). Ewes lambed normally and post mortem was conducted on the offspring at 4 months of age during which skeletal muscle samples were collected.**

**Donor ewes**

**100% MER**

**70% MER**

**Recipient ewes**

**100% MER**

***Ad lib***

**70% MER**

**CC**

**100% MER**

**100% MER**

**CR**

**HH**

**HR**

***Ad lib***

**100% MER**

**100% MER**

**Birth**

**d1**

**d 6-7**

**4 months**

**1 month**

**4 month old lambs**

**Gestation**

**Skeletal muscle collection at**

**post mortem for quantification of RNA & protein abundance**

**Nutritional regime**

**Super ovulation**

**Embryo transfer**

**Conception**

**Table S1in File S1: Oligonucleotide primer sequences for qRT-PCR analysis of IR, GLUT-4 and reference genes in skeletal muscle of postnatal lambs**

| **Gene Name**  **(GenBank accession no.)** | **Forward** | **Reverse** |
| --- | --- | --- |
| IR  ( Y16092) | 5’ CATCCCCAGAAAATCATCTTCAG3’ | 5’ CAAGGGCTCTGCGTTTCCT3’ |
| GLUT-4  (AY949177) | 5’GTGGCCATCTTTGGCTTCGTG3’ | 5’CGGCTGAGATCTGGTCAAAC3’ |
| RPLP0  (BT021080) | 5’caaccctgaagtgcttgacat3’ | 5’aggcagatggatcagcca3’ |
